# Impending Carotid Blowout Stabilization Using an LT-D Tube

**DOI:** 10.1155/2014/531561

**Published:** 2014-04-01

**Authors:** G. Desuter, A. Gregoire, Q. Gardiner, P. M. Francois

**Affiliations:** ^1^Department of Otolaryngology Head & Neck Surgery, Cliniques Universitaires Saint-Luc, Université Catholique de Louvain, 10 Avenue Hippocrate, 1200 Brussels, Belgium; ^2^Department of Otolaryngology Head & Neck Surgery, Ninewells Hospital, University of Dundee, Dundee DD1 4HN, UK; ^3^Department of Emergency Medicine, Cliniques Universitaires Saint-Luc, Université Catholique de Louvain and Hôpital National des Forces Armées, Brussels, Belgium

## Abstract

Adequate stabilization of a patient presenting with a carotid blowout is one of the most challenging issues an on-call ENT surgeon can be confronted with. Reducing the bleeding and securing the airway are essential before more definitive management. We present the case of a 72-year-old patient with head and neck cancer who arrived at the emergency room with a carotid blowout and who was successfully stabilized using a King LT-D ventilation tube.

## 1. Introduction

Carotid blowout (CB) represents one of the most challenging problems for those caring for patients with head and neck cancer. While the literature in English over the last decade offers an abundance of articles on the endovascular treatment of CB, little is written about the initial stabilization of the patient. Patient stabilization is, however, essential prior to any further attempt at definitive treatment.

## 2. Case Report

We present a case of impending CB successfully stabilized and eventually treated by using a King LT-D ventilation tube.

The 72-year-old patient had sustained a sudden and massive bleed from the mouth at home. He was able to call the emergency services and was taken immediately by ambulance to our tertiary hospital emergency room. Paramedics were able to gain venous access but were not able to intubate the patient due to severe trismus. Baseline measures on admission were heart rate: 90/min, blood pressure: 91/67 mmHg, respiratory rate: 19/min, and SpO2: 96%.

Physical examination revealed slow but active bleeding and large blood clots within the oral cavity. Flexible endoscopy revealed a huge clot within the oropharynx almost obstructing the airway. Only a small passage through this clot allowed the patient to breathe. Any attempt to remove the clot was followed by an increase in the active bleeding.

The patient was unable to communicate, but the medical record revealed a history of recent neck recurrence (classified T0N3M0) of a base of tongue squamous cell carcinoma classified pT1pN1 M0 initially treated by glossectomy and unilateral neck dissection followed by external radiotherapy. The recurrence had been treated with concomitant chemoradiotherapy four months earlier.

A neck CT Scan performed one month earlier had shown local recurrence with tumor necrosis and carotid artery involvement. No complimentary procedure had been proposed to the patient.

Shortly after admission the patient's respiratory distress and oral bleeding increased and the decision was made to stabilize the patient by endotracheal intubation and subsequent packing of the oropharynx. Three attempts to intubate the patient failed and caused movement of the clot that increased the oral bleeding. The presence of teeth, limitation of mouth opening, and stiffness of the neck were the principal causes of repeated esophageal intubation. Eventually a King LT-D tube, size 8, was introduced “blindly” into the esophagus and the double cuff inflated. Insertion of the tube immediately stopped the oropharyngeal bleeding and allowed for manual ventilation of the patient. Mechanical ventilation was still impossible due to high ventilation pressures. A subsequent percutaneous tracheotomy, using a Cook set, was performed under excellent conditions. The King LT-D tube was left in place acting as an intraluminal compression device.

The patient was transferred to the ICU for further follow-up. After 36 hours of compression and ventilation the King LT-D tube was gently removed, with the interventional radiology team on standby. No bleeding occurred and the patient was progressively weaned off the ventilator. Arteriography revealed complete thrombosis of the left external carotid artery with no active bleeding. The patient died peacefully three days later with no recurrence of the bleeding.

## 3. Discussion

Carotid blowout management has been widely discussed during the last decade in English language literature [1–3]. This is due both to technical improvements and to easier access to interventional radiology in many hospitals. Algorithms have been published that clarify CB management. They usually start with the “stabilization” step. Successfully completing this step not only saves the patient's life but also allows the emergency team a period during the patient's care process in order (a) to gain a better understanding of the patient's medical history and prognosis, (b) to transfer the patient to a room or setting that can provide endovascular diagnosis and care, whether reconstructive (stent) or obstructive (coils), and (c) in the case of palliative care patients to inform the patient's family about their relative's critical status or impending death.

Whether prophylactic stenting would present favorable cost/benefits ratio remains unclear. Likewise, the mortality rate related to CB stabilization failure has not been reported yet [4–6].

Carotid blowout has been classified in the past into three different categories: (a) threatened CB: no bleeding but clinical or radiological evidence suggesting imminence of CB, (b) impending CB: sentinel hemorrhage that either resolves spontaneously or with packing or pressure, and (c) acute CB: hemorrhage that cannot be stopped by packing or pressure.

This taxonomy is theoretical but of little clinical interest and thus not mentioned in more recent articles.

In the clinical situation a patient who is bleeding will either respond or not respond to efforts at stabilization. If they do not respond then they are likely to die before reaching the interventional radiology room, the operation room, or the intensive care unit.

Rapid and effective stabilization is therefore of key importance.

Three different clinical presentations can be found: the patient is bleeding through the skin (open neck bleeding), the patient is bleeding within the upper aerodigestive tract (intraluminal bleeding), or both (mixed bleeding). Likewise, bleeding can be categorized as being (a) out of control or (b) completely or partially reduced by clotting or other stabilization maneuvers.

Intraluminal bleeds that are out of control lead to rapid death. The other clinical presentations and their outcomes are summarized in Table 1.

While external compression can be performed by almost anyone, securing the airway and providing intraluminal compression can be difficult and require experienced staff. This is even truer for head and neck cancer patients who may potentially present with anatomical modifications, airway edema, impaired mouth opening, or postradiotherapy neck stiffness.

The use of the King LT-D tube allows these difficulties to be overcome.

**Figure 1:**
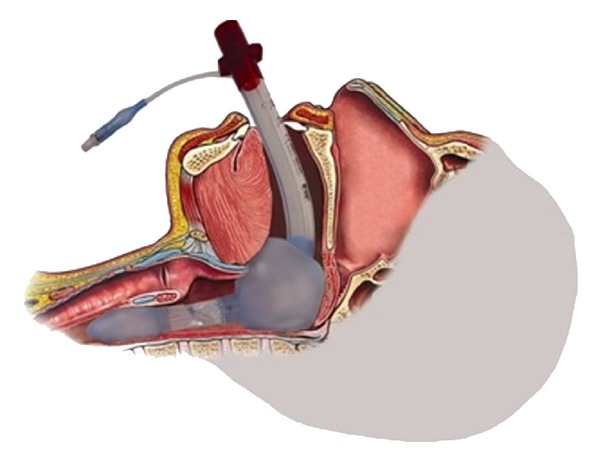
Anatomical drawing showing the King LT-D tube in place. Note the positioning of the esophageal and pharyngeal balloons (published with the authorization of VBM Medical Inc., Sulz, Germany).

The King LT-D tube (also called a supralaryngeal airway device) is a disposable and easily used alternative airway device that seals within the esophagus and the oropharynx to allow positive pressure ventilation (Figure 1). It is designed to be blindly inserted into the esophagus. The two cuffs are inflated through a single port with a 60 mL syringe. Ventilation occurs through multiple ventilation ports between the two cuffs. Its simplicity of use compared with an endotracheal intubation device has been demonstrated by several studies [7–11]. The LT-D tube is considered by many authors as an attractive device for expeditious airways management and indeed has become the most utilized ventilation device by paramedics in some US cities and in the US armed forces.

Only minor complications of its use, such as minor airway bleeding, odynophagia, or laryngeal spasm, have been published so far. One case of tongue engorgement has been associated with prolonged use of the LT-D tube, hypothetically related to compression of the lingual veins [12].

To our knowledge, this is the first case report of the use of the LT-D tube in CB in order to secure the airway and allow intraluminal compression with definitive cessation of bleeding after 36 hours.

Intubation was achieved on the first attempt despite the presence of trismus and neck stiffness. The low pressure oropharyngeal cuff, once inflated, immediately stopped the hemorrhage by sealing the pharynx.

**Figure 2:**
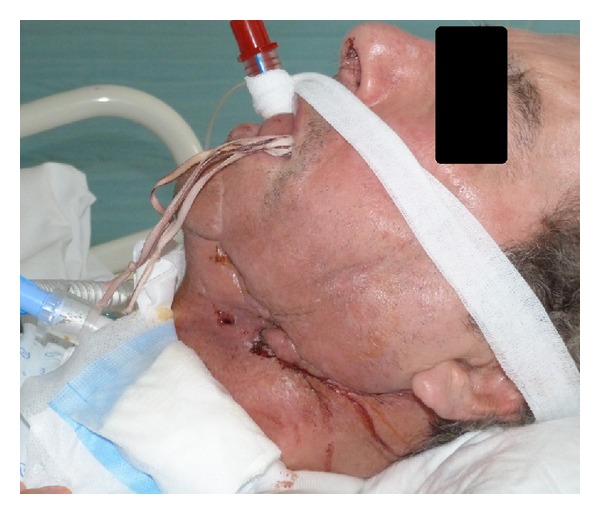
King LT-D tube in place acting as a pharyngeal compression device. Patient is ventilated through a tracheostomy.

In our case, mechanical ventilation through the LT-D required an elevated ventilation pressure. A second step percutaneous tracheotomy, performed in optimal conditions, allowed long term mechanical ventilation, while the LT-D tube was left in place to control the hemorrhage (Figure 2).

No further bleeding was noted after tube removal 36 hours later.

## 4. Conclusion

Insertion of the King LT-D tube offers adequate airway and circulatory stabilization in cases of intraluminal CB within the oropharynx. Its use is safe and efficient, requiring less skilled medical or paramedical staff. Stabilization of the patient then allows appropriate long term management of CB, whether conservative, endovascular, or surgical.

This case represents a new method for achieving CB stabilization. Further cases should be collected in order to assess its curative potential when compared to endovascular treatment.

## Figures and Tables

**Table 1:** Clinical presentations of CB and their outcome variables.

Type of CB	External compression	Outcome
Open neck bleeding	External compression possible	Outcome will depend on the intensity of the hemorrhage and the rapidity of compression

Intraluminal bleeding	External compression not relevant	Outcome will depend on the rapidity of securing the airway along with the ability to perform an intraluminal compression
